# *Staphylococcus aureus* - selective reporting of antibiogram results and its impact on antibiotic use: Interventional study with a reference group on the effect of switching from non-selective to selective antibiotic reporting

**DOI:** 10.1186/s13756-021-01021-7

**Published:** 2021-11-06

**Authors:** Franka Lestin-Bernstein, Ramona Harberg, Ingo Schumacher, Lutz Briedigkeit, Oliver Heese, Kristina Biedermann

**Affiliations:** 1 Department for Clinical Hygiene and Infectiology, Helios Clinics of Schwerin - University Campus of MSH Medical School Hamburg, Wismarsche Str. 393-397, 19055 Schwerin, Germany; 2 Central Pharmacy, Helios Clinics of Schwerin - University Campus of MSH Medical School Hamburg, Schwerin, Germany; 3 Anesthesiology and Intensive Care Medicine, Helios Clinics of Schwerin - University Campus of MSH Medical School Hamburg, Schwerin, Germany; 4 Institute for Laboratory and Transfusion Medicine, Helios Clinics of Schwerin - University Campus of MSH Medical School Hamburg, Schwerin, Germany; 5 Neurosurgery and Spinal Surgery, Helios Clinics of Schwerin - University Campus of MSH Medical School Hamburg, Schwerin, Germany; 6 Department for Clinical Hygiene and Infectiology, Helios Clinics of Schwerin - University Campus of MSH Medical School Hamburg, Schwerin, Germany

**Keywords:** Antimicrobial stewardship (AMS), *Staphylococcus aureus* infection, Selective reporting of susceptibility testing, Selective antibiogram, Recommended daily dose (RDD), Days of therapy (DOT), *Staphylococcus aureus* bacteremia (SAB)

## Abstract

**Background:**

Antimicrobial stewardship (AMS) strategies worldwide focus on optimising the use of antibiotics. Selective susceptibility reporting is recommended as an effective AMS tool although there is a lack of representative studies investigating the impact of selective susceptibility reporting on antibiotic use. The aim of this study was to investigate the impact of selective susceptibility reporting of *Staphylococcus aureus *(*S. aureus*) on antibiotic consumption. Enhancing the use of narrow-spectrum beta-lactam antibiotics such as flucloxacillin/cefazolin/cefalexin is one of the main goals in optimising antibiotic therapy of *S. aureus* infections.

**Methods:**

This interventional study with control group was conducted at a tertiary care hospital in Germany. During the one-year interventional period susceptibility reports for all methicillin-sensitive *S. aureus* (MSSA) were restricted to flucloxacillin/cefazolin/cefalexin, trimethoprim-sulfamethoxazole, clindamycin, gentamicin and rifampin/fosfomycin, instead of reporting all tested antibiotics. The impact of implementing selective reporting was analysed by monitoring total monthly antibiotic consumption in our hospital and in a reference hospital (recommended daily dose/100 occupied bed days: RDD/100 BD), as well as on an individual patient level by analysing days of therapy adjusted for bed days (DOT/ 100 BD) for patients with *S. aureus* bacteremia (SAB) and respectively skin and soft tissue infections (SSTI).

**Results:**

MSSA-antibiograms were acquired for 2836 patients. The total use of narrow-spectrum beta-lactams more than doubled after implementing selective reporting (from 1.2 to 2.8 RDD/100 BD, *P* < 0.001). The use of intravenous flucloxacillin/cefazolin for SAB rose significantly from 52 to 75 DOT/100 BD (plus 42%), just as the use of oral cefalexin for SSTI (from 1.4 to 9.4 DOT/100 BD, from 3 to 17 of 85/88 patients). Considering the overall consumption, there was no decrease in antibiotics omitted from the antibiogram. This was probably due to their wide use for other infections.

**Conclusions:**

As narrow-spectrum beta-lactams are not widely used for other infections, their increase in the overall consumption of the entire hospital was a strong indicator that selective reporting guided clinicians to an optimised antibiotic therapy of *S. aureus* infections. On a patient level, this assumption was verified by a significant improved treatment of *S. aureus* infections in the subgroups of SAB and SSTI. As useful AMS tool, we recommend implementing selective reporting rules into the national/international standards for susceptibility reporting.

**Supplementary Information:**

The online version contains supplementary material available at 10.1186/s13756-021-01021-7.

## Background

Significant efforts have been made to implement antimicrobial stewardship (AMS) worldwide to improve antibiotic prescribing to prevent multidrug resistance and improve patient care. There are a number of policies, strategies and tools outlined in different guidelines [1–3] and systematic reviews [4–9] to achieve this goal. Nevertheless, the impact of each tool is unclear.

One of the recommended tools is selective reporting of antibiotics in accordance with treatment guidelines to optimize antibiotic prescribing [1–3, 10–12]. Although this is one of the tools required there is a lack of representative studies investigating the impact of selective susceptibility reporting on antibiotic use.

*S. aureus* with its large number of pathogenicity factors causes severe infections that should be treated by optimal antibiotic therapy. Narrow-spectrum beta-lactam antibiotics such as flucloxacillin or cefazolin/cephalexin have better activity against *S. aureus* than broad-spectrum beta-lactams such as piperacillin-tazobactam, ceftriaxone or even cefuroxime [13–15]. In addition, third-generation cephalosporins or fluoroquinolones are associated with a number of side effects including *Clostridioides difficile* infections and the risk of selecting multi-resistant bacteria e.g. extended spectrum beta-lactamase-strains (ESBL) or methicillin-resistant *S. aureus* (MRSA). Therefore, enhancing the use of narrow-spectrum beta-lactams is one of the main goals of AMS in *S. aureus* infections to optimize antibiotic therapy of the individual patient and to prevent the spread of multi-resistant bacteria.

The aim of this study was to investigate the effect of switching from non-selective reporting of all tested antibiotics to selective reporting of recommended antibiotics in case of culturing *S.aureus*. Changes in other AMS tools were minimised during that period in order to focus on the impact of antibiotic reporting.

## Methods

This interventional study was conducted at the Helios Clinics of Schwerin, a tertiary care hospital in Germany with more than 1200 beds. Helios Clinic Duisburg, a tertiary care hospital of comparable size and structure (more than 1000 beds), served as a reference without intervention. The study was approved by the Ethics Committee of the Faculty of Medicine at the University of Rostock (A 2017-0149).

### Intervention

From November 01, 2017–October 31, 2018, reports on susceptibility testing (antibiogram) of all tested methicillin-sensitive *S. aureus* (MSSA) were modified in the following way: Only recommended therapeutically appropriate antibiotics for *S. aureus* infections were reported (narrow-spectrum beta-lactams: intravenous flucloxacillin/cefazolin/oral cefalexin, trimethoprim-sulfamethoxazole (TMP-SMX), clindamycin, gentamicin, rifampin/fosfomycin for combination therapy); whereas all others, especially broad-spectrum antibiotics (e.g. piperacillin-tazobactam, ceftriaxone, imipenem, meropenem, vancomycin) were excluded. The laboratory operation system (OPUS: L by OSM GmbH, Essen, Germany) was programmed to automatically omit these antimicrobials without operator intervention, in order to minimise effort and errors of the laboratory staff. The test results were still available on request. Before the intervention, all tested antibiotics were reported on the antibiogram, predetermined by industrial panels (Table 1). Additionally, the standard advice for the therapy of *S. aureus* infections was given in every susceptibility testing report to guide the clinician’s selection of the most appropriate antibiotic depending on the severity of the disease: "First choice for severe *S. aureus* infections/bacteremia: high dose intravenous flucloxacillin/cefazolin (ceftriaxone/cefotaxime/vancomycin are less effective in the treatment of MSSA); mild infection/oral follow-up: cefalexin, trimethoprim-sulfamethoxazole or clindamycin depending on the indication, side effects and allergies".

**Table 1:** Antibiogram for *S. aureus* prior to and after implementing selective reporting

Non-selective antibiogram reporting		Selective antibiogram reporting
Penicillin	Gentamicin	Oxacillin / Flucloxacillin iv
Ampicillin/Amoxicillin	Ciprofloxacin	
Piperacillin	Moxifloxacin	
Oxacillin / Flucloxacillin iv	Clindamycin	Cefazolin iv / Cefalexin po
Ampicillin-sulbactame	Erythromycin	
Amoxicillin-clavunate	Doxycycline	Trimethoprim-sulfamethoxazole
Piperacillin-tazobactam	Tigecyclin	
Cefazolin iv / Cefalexin po	Vancomycin	
Cefuroxime iv	Teicoplanin	Clindamycin
Cefotaxime	Daptomycin	
Ceftriaxone	Linezolid	Gentamicin
Ceftazidime	Trimethoprim-sulfamethoxazole	
Imipenem	Fosfomycin (combination therapy)	Fosfomycin (combination therapy)
Ertepenem	Rifampin (combination therapy)	Rifampin (combination therapy)

The prescribing clinicians were not informed of the ongoing study.

We did not change the reporting of susceptibility testing in methicillin-resistant *S. aureus* (MRSA).

### Measurement of the effect of implementing selective reporting and statistical analysis

#### Overall antibiotic consumption of the entire hospital

To measure the effect of the intervention we monitored the recommended daily dose/100 occupied bed days (RDD/100 BD) as a standardised method for measurement of antibiotic usage. To calculate RDD/100 BD, the total monthly use of every antibiotic in the entire hospital was devided by the occupied bed days and the assumed normal daily dose (Table 2: RDD for all involved antibiotics). Antibiotic consumption was compared to Helios Clinic Duisburg as a reference hospital of comparable size, where all tested antibiotics were furthermore reported on the antibiogram (antibiotic consumption data of all Helios Clinics in Germany are available on “iNAB": intranet-based statistics of antibiotic consumption). The RDD was representative of the actual dose of different antibiotics administered in both hospitals (besides dose adjustments to kidney or liver dysfunction). There were no temporary shortages of any involved antibiotics. The number of patients in whom *S. aureus* was detected was recorded monthly in order to have a baseline of infections for the intervention. For overall consumption of the entire hospital, the monthly use (RDD/100 BD) of each antibiotic was analysed by linear regression using an interrupted time-series approach with group comparisons (Table 3). In this analysis the outcome variable was analysed in dependence of the time (month) since start of study, a dummy variable for the intervention, a dummy variable for the clinic (interventional or reference clinic) and all interactions terms between these variables [16]. Simultaneously, we recorded the incidence of nosocomial *Clostridioides difficile* infections as recommended in the IDSA guideline [2] to observe a possible secondary effect of altered prescribing habits.

**Table 2:** Recommended daily dose (RDD) of the involved antibiotics

Antibiotic	Recommended daily dose (RDD in g)
Flucloxacillin iv	8
Ampicillin-sulbactam iv	6
Amoxicillin-clavunate po	1.75
Piperacillin-tazobactam iv	12
Imipenem-cilastatin iv	2
Meropenem iv	3
Cefalexin po	3
Cefuroxime iv	4,5
Cefuroxime po	1
Ceftriaxone iv	2
Trimethoprim-sulfamethoxazole iv/po	1.92/1.92
Clindamycin iv/po	1.8/1.8
Gentamicin iv	0.24
Ciprofloxacin iv/po	0.8/1
Moxifloxacin iv/po	0.4/0.4
Doxycycline iv/po	0.2/0.2
Vancomycin iv	2
Daptomycin iv	0.5
Linezolid iv/po	1.2/1.2
Fosfomycin iv	15
Rifampin iv/po	0.9/0.9

#### Antibiotic usage on an individual patient level

Based on the manual review of electronic medical records of our hospital, we additionally obtained individual patient level antibiotic use data for one year prior to and after implementing selective reporting.

We evaluated records of all patients with skin and soft tissue infections (SSTI) caused by *S. aureus* based on the German invoice system 'DRG' (diagnosis-related groups, group "L"). SSTI were chosen, because there had not been any prior AMS interventions in the relevant departments.

Furthermore we analysed records of all patients with *S. aureus* bacteremia (SAB) selected by the statistics program of the microbiology laboratory (HyBASE® by epiNET AG, Germany).

Days of therapy (DOT) were evaluated by three independent reviewers. They were counted commencing on day 2 after the receipt of the first *S. aureus* positive sample in the laboratory. This specific period was chosen; because this is the period it usually takes from the detection of *S. aureus* to a complete antibiogram being available in the electronic patient record in our hospital. For SAB, maximum follow up was 14 days per patient (recording lost to follow-up because of death, discharge or hospital transfer), as this is the recommended minimal duration of therapy in order to limit future antibiotic usage for secondary complications. Poisson regression was used to investigate the impact of implementing selective reporting on the individual patient level. The patient bed days (BD) were used as an exposure variable. The days of therapy / 100 bed days (DOT/100 BD) were estimated for each antibiotic in the period prior to and after the implementation of selective reporting. Incidence rate ratio (IRR) and corresponding p-values were calculated to compare DOT/100 BD in regard to the two periods.

Additionally, in case of SAB the number of patients with a "reasonable" therapy adaption (defined as replacing any other antibiotic by intravenous flucloxacillin or cefazolin within day 2 to 4) was counted prior to and after intervention.

Differences in patient characteristics (sex, secondary diagnoses / age, bed days) were tested by exact Fisher-test / Mann–Whitney-U-test. All statistical tests were two-sided and the significance level was set at 0.05. For statistical analysis, Stata/IC 16.1 for Unix was used (StataCorp 4905 Lakeway Drive College Station, Texas 77845 USA).

We refrained from any other AMS interventions during the interventional period, except for restricting the use of oral cefuroxime due to its insufficient absorption rate of approximately 50%. This was implemented in both clinics at the same time. In particular, there was no additional consultative support by infectious disease specialists in *S. aureus* infections.

## Results

The monthly number of patients with *S. aureus* detection in the Helios Clinics of Schwerin was not significantly different during the year prior to and after implementing selective reporting (117 vs. 99 patients per month, *p* = 0.844). That were approximately 0.5 patients with *S. aureus* infections/colonisations per 100 occupied bed days (BD), if the total occupied bed days of 549 511 during the observed two-year period is taken into account. The pre- and post-intervention antibiotic use of all available antibiotics in the Helios Clinics of Schwerin and the reference clinic is shown in Table 3.

**Table 3:** Antibiotic use one year prior to and after implementing selective reporting and change from prior to after implementation period in the Helios Clinics of Schwerin (S) and the reference clinic (R), estimated monthly use (RDD/100 BD), 95% CI

Antibiotics	Monthly use, year prior to* implementing selective reporting			Monthly use, year after* implementing selective reporting		Change of monthly use, after—prior *		
	S	R	Difference S-R**	S	R	S^#^	R^#^	Difference S-R^##^
*Selectively reported antibiotics*								
Flucloxacillin iv + Cefazolin iv	0.88 (0.75; 1.01)	0.75 (0.65; 0.85)	0.13 (−0.03; 0.30)	1.48 (1.29; 1.68)	0.94 (0.77; 1.10)	0.27 (−0.11; 0.65)	−0.45 (−0.81; −0.08)	0.72 (0.20; 1.23)
			*p* = 0.111			*p* = 0.158	***p* = 0.019**	***p* = 0.007**
Cefalexin po	0.29	0.19	0.10	1.35	0.27	1.01	−0.14	1.15
	(0.20; 0.38)	(0.05; 0.34)	(−0.07; 0.27)	(1.15; 1.55)	(0.19; 0.35)	(0.45; 1.56)	(−0.46; 0.17)	(0.53; 1.77)
			*p* = 0.239			***p* = 0.001**	p = 0.356	***p* = 0.001**
Trimethoprim-sulfamethoxazole	0.76 (0.54; 0.98)	2.21 (1.87; 2.56)	−1.45 (−1.87; −1.04)	1.12 (0.98; 1.27)	1.42 (1.19; 1.65)	0.14 (−0.40; 0.69)	−1.01 (−1.80; −0.22)	1.15 (0.22; 2.09)
			***p* < 0.001**			*p* = 0.590	***p* = 0.015**	***p* = 0.017**
Clindamycin iv/po	1.44 (1.26; 1.62)	1.27 (1.09; 1.45)	0.17 (−0.08; 0.43)	1.37 (1.13; 1.61)	1.05 (0.84; 1.25)	−0.20 (−0.91; 0.50)	−0.08 (−0.63; 0.46)	−0.12 (−0.98; 0.75)
			*p* = 0.177			*p* = 0.557	*p* = 0.751	*p* = 0.787
Sum of above named selectively reported antibiotics	3.38 (2.99; 3.77)	4.42 (3.85; 5.00)	−1.05 (−1.75; −0.35)	5.34 (4.97; 5.70)	3.67 (3.46; 3.88)	1.22 (−0.06; 2.49)	−1.68 (−2.89; −0.48)	2.90 (1.21; 4.60)
			***p* = 0.005**			*p* = 0.060	***p* = 0.008**	***p* = 0.001**
*Combination therapy only*								
Gentamicin iv	0.41 (0.32; 0.50)	0.28 (0.16; 0.40)	0.13 (−0.04; 0.30)	0.40 (0.26; 0.54)	0.58 (0.19; 0.98)	0.13 (−0.07; 0.34)	0.18 (−0.50; 0.86)	−0.05 (−0.73; 0.64)
			*p* = 0.119			*p* = 0.185	*p* = 0.587	*p* = 0.891
Rifampin iv/po	0.57 (0.42; 0.73)	0.31 (0.23; 0.38)	0.27 (0.09; 0.44)	0.59 (0.37; 0.81)	0.50 (0.30; 0.70)	0.18 (−0.32; 0.68)	0.23 (−0.14; 0.59)	−0.05 (−0.65; 0.55)
			***p* = 0.004**			*p* = 0.462	*p* = 0.206	*p* = 0.867
Fosfomycin iv	0.03 (0.01; 0.06)	0.00 (−0.00; 0.01)	0.03 (0.01; 0.05)	0.04 (0.01; 0.07)	0.02 (−0.01; 0.05)	0.00 (−0.06; 0.06)	−0.02 (−0.06; 0.01)	0.03 (−0.04; 0.09)
			***p* = 0.017**			*p* = 0.978	*p* = 0.126	*p* = 0.426
*No longer reported antibiotics*								
Ampicillin-sulbactam + Amoxicillin-clavunate iv/po	5.49 (4.97; 6.01)	4.28 (3.91; 4.65)	1.21 (0.57; 1.86)	5.77 (5.38; 6.15)	4.45 (4.00; 4.89)	1.34 (0.27; 2.40)	0.15 (−0.96; 1.25)	1.19 (−0.29; 2.67)
			***p* = 0.001**			***p* = 0.016**	*p* = 0.786	*p* = 0.113
Piperacillin-tazobactam iv	3.91 (3.56; 4.25)	5.57 (5.08; 6.07)	−1.66 (−2.28; −1.04)	4.50 (4.09; 4.91)	5.86 (5.43; 6.30)	0.56 (−0.42; 1.55)	1.08 (−0.15; 2.30)	−0.51 (−2.04; 1.01)
			***p* < 0.001**			*p* = 0.247	*p* = 0.082	*p* = 0.501
Cefuroxime iv/po	6.16 (5.86; 6.46)	3.97 (3.68; 4.25)	2.20 (1.78; 2.61)	3.77 (3.55; 3.99)	2.21 (1.88; 2.55)	−0.53 (−1.18; 0.13)	−0.43 (−1.32; 0.45)	−0.09 (−1.16; 0.97)
			***p* < 0.001**			*p* = 0.110	*p* = 0.321	*p* = 0.860
Ceftriaxone iv	3.39 (3.09; 3.68)	2.96 (2.58; 3.35)	0.42 (−0.09; 0.93)	3.75 (3.38; 4.11)	3.10 (2.58; 3.62)	−0.22 (−1.11; 0.68)	0.70 (−0.73; 2.14)	−0.92 (−2.56; 0.72)
			*p* = 0.101			*p* = 0.618	*p* = 0.318	*p* = 0.262
Imipenem-cilastatin iv + Meropenem iv	1.97 (1.74; 2.20)	2.43 (2.23; 2.62)	−0.46 (−0.76; −0.15)	2.35 (2.10; 2.60)	2.30 (2.01; 2.59)	0.29 (−0.48; 1.06)	−0.63 (−1.26; −0.01)	0.92 (−0.03; 1.88)
			***p* = 0.005**			*p* = 0.440	***p* = 0.047**	*p* = 0.059
Ciprofloxacin iv/po	3.37 (3.01; 3.73)	4.09 (3.64; 4.55)	−0.72 (−1.40; −0.05)	3.56 (3.29; 3.83)	3.48 (3.02; 3.93)	−0.50 (−1.48; 0.48)	−0.71 (−1.69; 0.26)	0. 21 (−1.12; 1.55)
			***p* = 0.038**			*p* = 0.298	*p* = 0.142	*p* = 0.749
Vancomycin iv	0.97 (0.85; 1.08)	1.69 (1.48; 1.90)	−0.73 (−0.97; −0.48)	0.94 (0.80; 1.09)	1.58 (1.41; 1.76)	0.37 (0.06; 0.68)	−0.32 (−0.92; 0.27)	0.69 (0.05; 1.34)
			***p* < 0.001**			*p* = 0.023	*p* = 0.266	***p* = 0.036**
Linezolid iv/po	0.37 (0.28; 0.46)	0.43 (0.33; 0.52)	−0.05 (−0.19; 0.09)	0.39 (0.32; 0.45)	0.37 (0.31; 0.43)	−0.17 (−0.36; 0.03)	0.01 (−0.29; 0.31)	−0.18 (−0.53; 0.17)
			*p* = 0.442			*p* = 0.097	*p* = 0.943	*p* = 0.315
Doxycycline iv/po	0.50 (0.23; 0.77)	0.22 (0.10; 0.34)	0.28 (−0.01; 0.58)	0.67 (0.56; 0.79)	0.58 (0.44; 0.73)	−0.04 (−0.56; 0.48)	0.00 (−0.34; 0.34)	−0.04 (−0.64; 0.57)
			*p* = 0.061			*p* = 0.882	*p* = 1.000	*p* = 0.901
Daptomycin iv	0.04 (0.01; 0.08)	0.01 (−0.00; 0.03)	0.03 (−0.01; 0.07)	0.10 (0.04; 0.16)	0.02 (−0.00; 0.04)	0.08 (−0.03; 0.20)	−0.04 (−0.10; 0.02)	0.13 (0.00; 0.26)
			*p* = 0.124			*p* = 0.148	*p* = 0.136	***p* = 0.047**
Sum of above named no longer reported antibiotics	42.36 (40.59; 44.14)	42.42 (40.17; 44.66)	−0.05 (−2.97; 2.87)	44.47 (42.87; 46.07)	40.69 (38.87; 42.51)	5.34 (−0.06; 10.74)	1.06 (−4.33; 6.44)	4.28 (−3.11; 11.67)
			*p* = 0.971			*p* = 0.052	*p* = 0.686	*p* = 0.249
Total antibiotic use iv/po	43.25 (41.45; 45.04)	43.16 (40.89; 45.44)	0.08 (−2.88; 3.04)	45.96 (44.37; 47.54)	41.62 (39.70; 43.55)	5.61 (0.24; 10.97)	0.61 (−5.04; 6.26)	5.00 (−2.56; 12.55)
			*p* = 0.955		***p* = 0.041**	*p* = 0.824	*p* = 0.189	
Incidence of nosocomial infections with *Clostridioides difficile* (*C. difficile* associated cases / 100 total cases)	0.09 (0.06; 0.11)	0.30 (0.21; 0.38)	−0.21 (−0.30; −0.12)	0.04 (0.02; 0.06)	0.21 (0.16; 0.26)	−0.03 (−0.10; 0.03)	0.07 (−0.11; 0.24)	−0.10 (−0.28; 0.08)
			***p* < 0.001**			*p* = 0.289	*p* = 0.451	*p* = 0.281

*RDD* recommended daily dose, *BD* bed days

^*^Estimation by interrupted time series analysis using linear regression

^**^Estimated difference between Schwerin (interventional clinic) and reference clinic, prior implementing selective reporting

^#^Estimated difference of monthly antibiotic use prior to and after implementing selective reporting (after—prior), *p*-value (null hypothesis: no change from prior to after intervention)

^##^Estimated difference (95%-CI) between intervention and reference clinic with respect to the difference of monthly use from prior to after intervention period, *p*-value (null hypothesis: the mean difference from prior to after intervention is the same in both clinics, *p*-values < 0.05 in bold)

### Was there any impact of selective susceptibilitiy reporting on the antibiotic consumption of the entire hospital (Table 3, Figs. 1 and 2)?

**Fig. 1:**
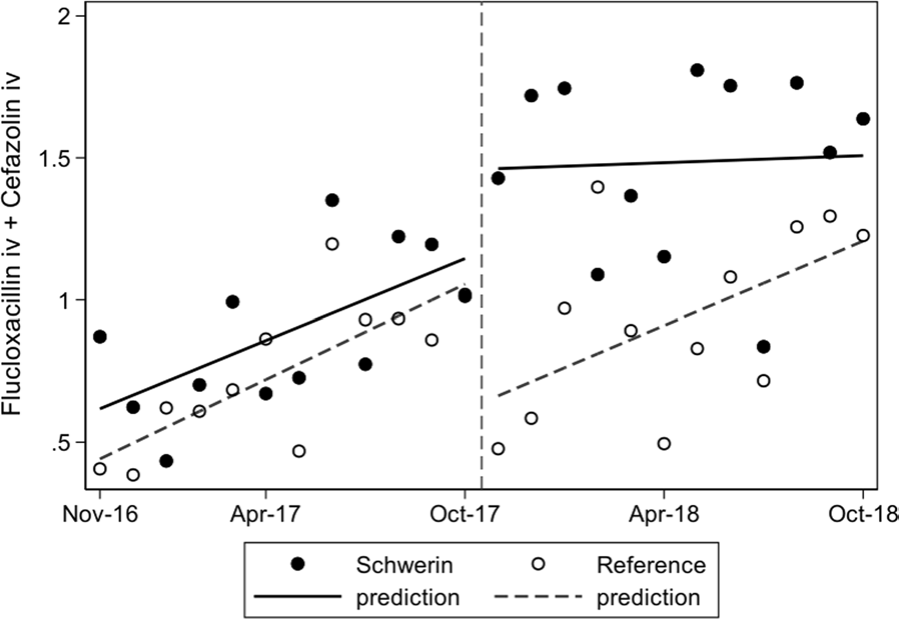
Monthly use (RDD/100 BD) of intravenous small-spectrum beta-lactams prior to and after implementing selective reporting in the Helios Clinics of Schwerin and the reference clinic; actual values and linear prediction by interrupted time series analysis

**Fig. 2:**
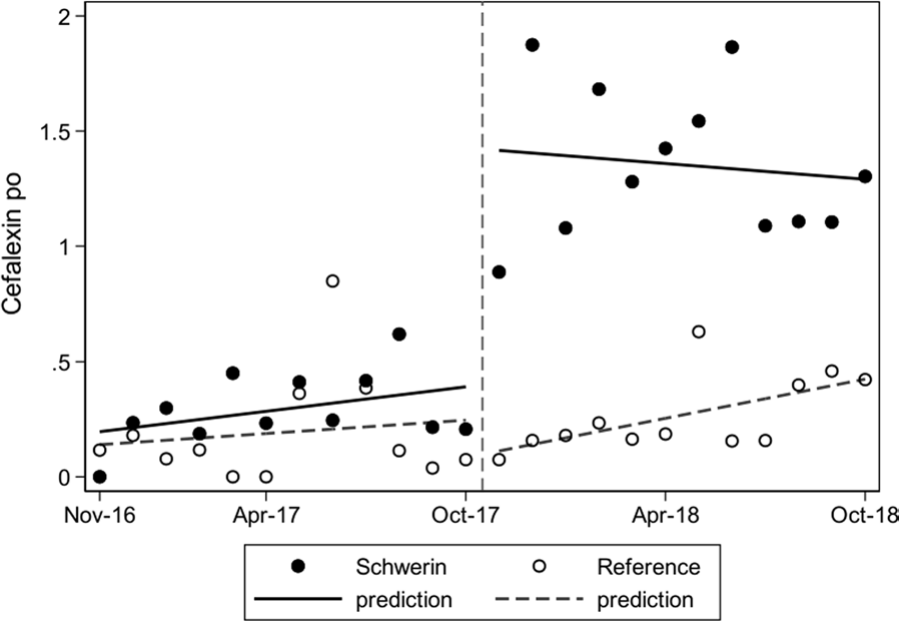
Monthly use (RDD/100 BD) of oral Cefalexin prior to and after implementing selective reporting in the Helios Clinics of Schwerin and the reference clinic; actual values and linear prediction by interrupted time series analysis

Before the start of selective reporting, no difference in the total consumption of the narrow-spectrum beta-lactams (flucloxacillin/cefazolin/cefalexin) could be found between the two hospitals (Table 3). After the implementation of selective reporting in the interventional clinic, there was a significant increase in their total use. This increased use was detected for both intravenous flucloxacillin and cefazolin as first line treatment for severe *S. aureus* infections (from 0.88 to 1.48 RDD/100 BD, *p* = 0.007), as well as use of oral cefalexin for less severe infections or follow-up therapy (from 0.29 to 1.35 RDD/100 BD, *p* = 0.001). Furthermore, an increase in the use of trimethoprim-sulfamethoxazole (*p* < 0.017) was detected after selective reporting was introduced. Overall, there was a statistically significant increase in the usage of selectively reported antibiotics in the interventional clinic (from 3.38 to 5.34 RDD/100 BD, *p* = 0.001).

Regarding the course of time there was a slight monthly increase of intravenous flucloxacillin/cefazolin usage in both clinics, but after implementing selective reporting in the interventional clinic the consumption rose immediately and consolidated on a higher level (Fig. 1). For oral cefalexin the interrupted time series analysis showed an immidiate and sustained higher monthly use in contrast to the reference clinic (Fig. 2).

Regarding the antibiotics omitted from the report, no significant change in the consumption was observed after intervention (Table 3). The only exception to this was a slight increase in the use of vancomycin and the very rarely used daptomycin. Compared to the reference clinic, there was a significant lower use of vancomycin and carbapenems in our clinic before the intervention, which aligned to a comparable level of use the year after. Carbapenems were analysed in sum as imipenem was replaced by meropenem during the investigation period due to its lower risk of seizures and expiring patent. For that reason, clinicians were forced to use meropenem instead of imipenem in most indications (e.g. calculated sepsis therapy).

Considering the total overall antibiotic use, there was no relevant difference between boths clinics prior to and after intervention. The incidence of nosocomial *C. difficile* infections was significantly lower in our clinic even before intervention, without a relevant change after intervention.

### Was there any impact of selective susceptibility reporting on the antibiotic treatment of skin and soft tissue infections (SSTI) on an individual patient level (Table 4)?

**Table 4:** Skin and soft tissue infections (SSTI) caused by *S. aureus*—estimated days of therapy per 100 bed days: one year prior to and one year after implementing selective reporting

Antibiotics	Prior to implementing elective reporting n = 85, BD = 729	After implementing selective reporting n = 88, BD = 562	DOT per 100 BD after vs. prior^**#**^	
	pat/ DOT/DOT per 100 BD (95%-CI)*	pat/ DOT/DOT per 100 BD (95%-CI)*	IRR (95%-CI)	*p*
*Selectively reported antibiotics*				
Flucloxacillin iv + Cefazolin iv	9 / 61 / 8.4 (2.4; 14.4)	18 / 101 / 18.0 (10.3; 25.6)	2.15 (0.93; 4.95)	0.073
Cefalexin po	3 / 10 / 1.4 (−0.3; 3.1)	17 / 53 / 9.4 (4.5; 14.4)	6.87 (1.81; 26.15)	**0.005**
Trimethoprim-sulfamethoxazole	2 / 3 / 0.4 (−0.2; 1.0)	0	0	–
Clindamycin iv/po	12 / 73 / 10.0 (3.3; 16.7)	15 / 61 / 10.9 (4.6; 17.1)	1.08 (0.45; 2.61)	0.857
Sum of above named selectively reported antibiotics	21 / 147 / 20.2 (11.0; 29.3)	36 / 215 / 38.3 (27.2; 49.3)	1.90 (1.11; 3.25)	**0.020**
*Combination therapy only*				
Gentamicin + Tobramycin + Amikacin iv	0 / 0 / 0 (0.0; 0.0)	1 / 4 / 0.7 (−0.7; 2.1)	–	–
Rifampin iv/po	1 / 3 / 0.4 (-0.4; 1.2)	1 / 4 / 0.7 (−0.7; 2.1)	1.73 (0.11; 28.23)	–
Fosfomycin iv	1 / 2 / 0.3 (−0.3; 0.8)	0	0	–
*No longer reported antibiotics*				
Penicillin G + Ampicillin/Amoxicillin iv	2 / 10 / 1.4 (−0.7; 3.4)	4 / 14 / 2.5 (−0.0; 5.0)	1.82 (0.30; 11.14)	–
Ampicillin-sulbactam + Amoxicillin-clavunate iv/po	42 / 173 / 23.7 (14.7; 32.7)	56 / 192 / 34.2 (23.7; 44.7)	1.44 (0.88; 2.35)	0.143
Piperacillin-tazobactam	5 / 13 / 1.8 (−0.2; 3.8)	7 / 37 / 6.6 (1.6; 11.5)	3.69 (0.97; 14.03)	0.055
Cefuroxime iv/po	24 / 95 / 13.0 (7.2; 18.9)	14 / 57 / 10.1 (3.7; 16.6)	0.78 (0.36; 1.69)	0.527
Ceftriaxone iv + Ceftazidime iv + Cefpodoxime po	2 / 27 / 3.7 (-2.2; 9.6)	0	0	–
Imipenem-cilastatin iv + Meropenem iv	4 / 27 / 3.7 (−0.3; 7.7)	0	0	–
Ciprofloxacin + Levofloxacin + Moxifloxacin iv/po	8 / 27 / 3.7 (0.7; 6.7)	2 / 10 / 1.8 (−0.8; 4.3)	0.48 (0.09; 2.52)	0.385
Sum of above named no longer reported antibiotics	75 / 372 / 51.0 (40.8; 61.2)	76 / 310 / 55.2 (45.7; 64.6)	1.08 (0.83; 1.41)	0.562

^*^Pat/ DOT/DOT per 100 BD (95%-CI): number of patients, days of therapy (DOT), days of therapy per 100 bed days (BD) with 95%-confidence interval estimated by Poisson regression;

^#^Poisson regression, incidence rate ratio (IRR) with 95%-confidence interval (CI) and *p*-value (*p*), *p*-values < 0.05 in bold. In case of less than 5 patients in both periods no statistical test was performed

Searching the DRG system for SSTI "and" *S. aureus*, we found 222 patients in the year before and 190 patients in the year after intervention. Excluding all patients with MRSA infection, colonization without treatment, mixed infection with other pathogens or death/discharge before susceptibility testing (day 2), the data of 85 versus 88 patients was analysed (Additional file 1: Flow chart exclusion criteria SSTI). The groups were inhomogeneous regarding age (63 versus 55 years in median), comorbidities (chronic renal failure 25 versus 10%) and length of hospital stay (6 versus 3 days in median).

Comparing days of therapy (DOT) of different antibiotics before and after implementing selective reporting (Table 4), we found more than a doubling of DOT per 100 bed days (BD) from 8.4 to 18.0 in the use of intravenous flucloxacillin/cefazolin (primarily used for severe SSTI only). However, this was not statistically significant. The use of intravenous cefazolin was established in our clinic for the first time (data not shown). There was a significant, almost sevenfold increase in the use of oral cefalexin (from 1.4 to 9.4 DOT/100 BD, *p* = 0.005), without a concurrent reduction in the total use of cefuroxime (13.0 vs. 10.1, *p* = 0.527)—despite the withdrawal of oral cefuroxime. In sum, the use of all selectively reported antibiotics nearly doubled from 20 to 38 DOT/100 BDs, reaching statistical significance. In contrast, the use of third-generation cephalosporins and carbapenemes ceased completely.

### Was there any impact of selective susceptibility reporting on the antibiotic treatment of* S. aureus* bacteremia (SAB) on an individual patient level (Table 5)?

**Table 5:** *S. aureus* bacteremia (SAB)—estimated days of therapy per 100 bed days: one year prior to and one year after implementing selective reporting

Antibiotics	Prior to implementing selective reporting n = 86, BD = 943	After implementing selective reporting n = 81, BD = 946	DOT per 100 BD after vs. prior to^**#**^	
	pat/ DOT/DOT per 100 BD (95%-CI)*	pat/ DOT/DOT per 100 BD (95%-CI)*	IRR (95%-CI)	*p*
Selectively reported antibiotics				
Flucloxacillin iv + Cefazolin iv	50 / 494 / 52.4 (42.8; 62.0)	72 / 706 / 74.6 (67.5; 81.8)	1.42 (1.16; 1.75)	**0.001**
Cefalexin po	2 / 5 / 0.5 (−0.2; 1.3)	3 / 9 / 1.0 (−0.3; 2.2)	1.79 (0.27; 12.01)	–
Trimethoprim-sulfamethoxazole	1 / 3 / 0.3 (−0.3; 0.9)	2 / 5 / 0.5 (−0.3; 1.4)	1.66 (0.13; 20.94)	–
Clindamycin iv/po	8 / 47 / 5.0 (0.9; 9.0)	6 / 28 / 3.0 (−0.3; 6.2)	0.59 (0.15; 2.34)	0.457
Sum of above named selectively reported antibiotics	53 / 549 / 58.2 (48.4; 68.1)	74 / 748 / 79.1 (71.4; 86.7)	1.36 (1.12; 1.65)	**0.002**
Combination therapy only				
Gentamicin + Tobramycin + Amikacin iv	6 / 32 / 3.4 (0.3; 6.5)	5 / 29 / 3.1 (0.3; 5.8)	0.90 (0.25; 3.28)	0.877
Rifampin iv/po	15 / 124 / 13.1 (6.6; 19.7)	9 / 92 / 9.7 (3.3; 16.2)	0.74 (0.32; 1.70)	0.476
Fosfomycin iv	2 / 8 / 0.8 (−0.5; 2.2)	1 / 7 / 0.7 (−0.7; 2.2)	0.87 (0.07; 10.47)	–
No longer reported antibiotics				
Penicillin G + Ampicillin/Amoxicillin iv	2 / 11 / 1.2 (−0.4; 2.8)	1 / 1 / 0.1 (−0.1; 0.3)	0.09 (0.01; 1.00)	–
Ampicillin-sulbactam + Amoxicillin-clavunate iv/po	18 / 69 / 7.3 (3.3; 11.3)	2 / 4 / 0.4 (−0.2; 1.0)	0.06 (0.01; 0.25)	** < 0.001**
Piperacillin-tazobactam	27 / 128 / 13.6 (7.6; 19.5)	34 / 158 / 16.7 (10.4; 23.0)	1.23 (0.69; 2.19)	0.481
Cefuroxime iv/po	10 / 68 / 7.2 (2.2; 12.2)	2 / 14 / 1.5 (−1.0; 4.0)	0.21 (0.03; 1.28)	0.090
Ceftriaxone iv + Ceftazidime iv + Cefpodoxime po	10 / 46 / 4.9 (1.3; 8.5)	10 / 28 / 3.0 (0.5; 5.4)	0.61 (0.20; 1.84)	0.378
Imipenem-cilastatin iv + Meropenem iv	11 / 49 / 5.2 (1.6; 8.8)	14 / 69 / 7.3 (3.2; 11.4)	1.40 (0.58; 3.42)	0.455
Ciprofloxacin + Levofloxacin + Moxifloxacin iv/po	14 / 78 / 8.3 (3.7; 12.9)	12 / 79 / 8.4 (3.1; 13.6)	1.01 (0.44; 2.33)	0.982
Vancomycin iv	13 / 44 / 4.7 (1.8; 7.5)	7 / 31 / 3.3 (−0.3; 6.8)	0.70 (0.20; 2.43)	0.577
Linezolid iv/po	2 / 13 / 1.4 (−0.9; 3.7)	2 / 8 / 0.8 (−0.6; 2.3)	0.61 (0.06; 6.76)	–
Sum of above named no longer reported antibiotics	70 / 506 / 53.7 (44.6; 62.7)	58 / 392 / 41.4 (31.4; 51.5)	0.77 (0.57; 1.04)	0.087
Patients with therapy adaption to intravenous flucloxacillin/cefazolin on day 2–4, n (%)	36 (41.9%)	62 (76.5%)		**< 0.001** ^**##**^

Follow up to a maximum of 14 bed days per patient (except early exit because of death, discharge or hospital transfer)

^*^Pat/ DOT/DOT per 100 BD (95%-CI): number of patients, days of therapy (DOT), days of therapy per 100 bed days (BD) with 95%-conifdence interval estimated by Poisson regression;

^#^Poisson regression, incidence rate ratio (IRR) with 95%-confidence interval (CI) and *p*-value (*p*), *p*-values < 0.05 in bold. In case of less than 5 patients in both periods no statistical test was performed

^##^Exact Fisher-test

Searching the Hybase program we found 96 patients with SAB (MRSA excluded) in the year prior to versus 98 patients after implementing selective reporting. All pediatric patients and patients dead, discharged or transferred to another hospital before day 2 were excluded, as were all patients with missing medical records or allergy to penicillin (Additional file 2: Flow chart exclusion criteria SAB). Therefore we analysed the records of 86 versus 81 patients, including 7 versus 16 patients with additional further infections. There were no significant differences of both groups in terms of sex (71 vs. 59% male), age (72 vs. 70 years in median), comorbidities, treating specialty or rate of death within 14 days of therapy (14 vs. 15%).

We found a significant higher use of intravenous flucloxacillin/cefazolin (recommended standard therapy for SAB) after implementing selective reporting, rising from 52.4 to 74.6 DOT/100 BD (+ 42%). The sum of therapy days of all selectively reported antibiotics (except antibiotics for combination therapy only) rose in a similar way. In exchange, there was a sharp decline of the no longer reported ampicillin-sulbactam/amoxicillin-clavunate from 69 to 4 DOT/100 BD and a trend towards lower prescription of most of the other not reported antibiotics (incidence rate ratio 0.77, *p* = 0.087).

The number of appropriate adaptions of the antibiotic treatment of SAB significantly rose from approximately 42 to 77% of cases (solely the exchange from any other to intravenous flucloxacillin or cefazolin was considered "appropriate"). The total proportion of patients on flucloxacillin/cefazolin rose from 58 to 89%, including all patients who had initially received flucloxacillin.

As a measure of antibiotic consumption, we additionally calculated all DOT per 1000 admissions for SAB and SSTI—without any additional information.

## Discussion

Is it possible to guide clinicians to prescribe the optimal antibiotic therapy for *S. aureus* infections by solely reporting the most effective antibiotics on the antibiogram? A consulting infectious disease specialist (ID)/clinical microbiologist has a huge impact on the optimized therapy of *S. aureus* infections, but this is costly and often not possible [2, 3, 6, 17, 18]. Guiding the clinician using the antibiogram as an AMS tool could be a very cost-effective alternative, even if it cannot fully replace an ID consultation. Selective antibiotic reporting is recommended by most AMS guidelines [1–3, 12, 19, 20], although the evidence is very scant: very few studies have proved a significant effect on antibiotic consumption for urinary tract infections or infections due to gram-negative pathogens [21–25] or for the use of rifampicin [26]. To our knowledge, there are no studies on frequently occurring and often severe *S. aureus* infections. To date, selective reporting is poorly implemented in Europe (only in about one third of European countries), predominantly for urine cultures; only in Ireland, Turkey, the UK and Sweden is it endorsed as a standard of care by the health care authorities [19, 20, 27–29].

### Which antibiotics are the most effective in *S. aureus* infections to be reported on the "selective" antibiogram?

There is broad consensus that narrow-spectrum beta-lactams such as intravenous flucloxacillin or first generation cephalosporins (cefazolin/cefalexin) have better activity against MSSA than broad-spectrum beta-lactams. The treatment of bacteremia caused by *S. aureus* with high dose intravenous flucloxacillin or cefazolin is associated with lower mortality rates compared to the treatment with broad-spectrum beta-lactams or vancomycin [13–15, 30–35]. There is a minimum consensus amongst publications to report oxacillin/flucloxacillin, clindamycin and trimethoprim/ trimethoprim-sulfamethoxazole (the latter for oral treatment) on a selective antibiogram for *S. aureus*, as well as to omit vancomycin, linezolid or broad-spectrum beta-lactams [19, 20, 36].

### How can we measure the effect of the intervention?

We monitored the monthly consumption of different antibiotics using RDD/100 BD, a standardised method for measuring antibiotic use, which is not influenced by fluctuating patient numbers. We compared consumption in the year prior to and after implementing selective susceptibility reporting in our clinic to the reference clinic. We used RDD instead of the frequently used defined daily dose (DDD) [2, 37] as the RDD is based on higher daily doses. This represents the doses used in our investigation more closely (Table 2).

Additionally, we analysed antibiotic use on an individual patient level using “Days of therapy” (DOT), favoured by IDSA guideline 2016 [2], as this is not impacted by individual dose adjustments. As our electronic patient file system does not allow an automatic assessment of DOT, patient records were analysed manually by three independent reviewers. Thus, we focussed on two patient groups: SSTI and SAB. We chose SSTI because—in contrast to other specialties—there had not been any previous AMS interventions in the involved departments.

### What was the impact of the intervention on the use of selectively reported antibiotics?

Regarding the overall consumption of the hospital there was a significant increase of antibiotics recommended for *S. aureus* infections (from 3.38 to 5.34 RDD/100 BD, *p* = 0.001; Table 3) after selectively reporting commenced. This increase was particularly remarkable when put into proportion to the overall low rate of *S. aureus* infections (less than 0.5 newly detected *S. aureus* infections/colonisations/100 BD).

Especially the use of narrow-spectrum beta-lactams (flucloxacillin/cefazolin/cefalexin) rose significantly after selective reporting.

Intravenous flucloxacillin and cefazolin—used in SAB and the initial therapy of severe *S. aureus* infections—were analysed as an entity because they were assessed to be equally effective and replaced each other depending on side effects [30, 31, 38]. Their consumption rose from 0.9 to 1.5 RDD/100 BD throughout the hospital. Although a slight increase in the overall use of narrow spectrum beta-lactams could be seen in both hospitals over the course of time (probably due to general AMS interventions in Germany), the changing of antibiograms in our clinic had an immediate and sustained effect on the treatment of SAB in contrast to the reference clinic (Fig. 1).

There was also an immediate striking increase in the consumption of oral cefalexin used in milder infections or follow-up therapy (from 0.3 to 1.4 RDD/100 BD, *p* = 0.001) (Table3, Fig. 2). This increase exceeds a possible regulatory effect due to a restriction of oral cefuroxime, preauthorised by the pharmacy (simultaneously in both clinics; plus 1.01 oral cefalexin versus minus 0.53 RDD/100 BDD total cefuroxime in the interventional clinic).

As the use of narrow-spectrum beta-lactams was restricted to targeted treatment of *S.aureus* infections in both hospitals, the overall consumption data indicates that the selective antibiogram significantly increased their use for those infections.

This assumption was confirmed by our individual patient level data. Days of therapy (DOT) of all selectively reported antibiotics rose significantly from 20 to 38 DOT/100 BD (*p* = 0.020) in SSTI (Table 4) and from 58 to 79 DOT/100 BD (*p* = 0.002) in SAB (Table 5). In SSTI, we recorded a striking rise of oral cefalexin usage (predominantly used for mild infections without bacteremia) from 1.4 to 9.4 DOT/100 BD (*p* = 0.005; from 3 to 17 patients), whereas in SAB there was a significant increase in the use of intravenous flucloxacillin/cefazolin from 52 to 75 DOT/100 BD (*p* = 0.001; from 50 to 72 patients). Selective reporting obviously strongly supported clinicians to optimize the treatment of *S. aureus* infections after receiving the report. In 77% of cases with SAB, the therapy was converted to a flucloxacillin/cefazolin regime on day 2–4. This is in contrast to 42% conversion rate before the introduction of selective reporting. If you include cases where treatment had initially been started on a flucloxacillin/cefazolin regime, 72 of 81 (89%) of the patients with SAB received appropriate treatment (50 of 86 before intervention). This number could hardly be further increased, since amongst the SAB group there were patients requiring broader therapy spectrum due to further infections (7 vs. 16 patients, mainly aspiration pneumonias or urinary tract infections). We did not exclude these patients because they were difficult to determine (proven versus suspected infections).

Tan et al.[21] showed a similar significant increase in the consumption of selectively reported antibiotics such as nitrofurantoin for targeted therapy and even for calculated therapy of urinary tract infections. Also, for urinary tract infections McNulty et al. [23]*.* demonstrated "that prescribing reverted to pre-intervention levels once the change in antibiotic reporting had stopped". We decided not to revert the antibiograms to the pre-interventional stage due to ethical reasons, and the goal to enhance the use of more effective narrow-spectrum antibiotics, along with lowering side-effects was reached.

### What was the impact of selective reporting on the use of omitted antibiotics?

Considering the overall consumption of the hospital (RRD/100 BD, Table 3), there was no decrease in the use of wide-spectrum antibiotics after selectively not-reporting for *S. aureus* (42.4 vs. 44.5 RDD/100 BD, *p* = 0.249). Neither was there an impact on the number of *C. difficile infections*. We didn´t expect this anyways due to the wide use of these broader spectrum antibiotics for other infections, e.g. sepsis, pneumonia or meningitis. There was even a slight (partly significant) rise in the usage of broad-spectrum antibiotics such as carbapenems and vancomycin in our clinic. In effect this led to an aligning with the significantly higher baseline level of piperacillin-tazobactam, carbapenems, fluorochinolons and vancomycin usage reported by the reference clinic for the pre-interventional period (see also limitations of RDD/100 BD).

Due to the overall low proportion of *S.aureus* infections, the reduction in the use of omitted antibiotics might be concealed due to their higher usage for other infections. We therefore evaluated individual patient records for two specific indications additionally. In the SAB group (Table 5), there was a significant decline in the use of aminopenicillin-beta-lactamase-inhibitors (from 7.3 to 0.4 DOT/100 BD, from 18 to 2 patients), in favour of intravenous flucloxacillin/cefazolin. A trend towards lower prescription rates of all no longer reported antibiotics (from 54 to 41 DOT/100 BD, *p* = 0.087) did not reach statistical significance however. This was probably due to low case numbers (86 vs. 81) and polymicrobial infections (see above). In the SSTI group (Table 4), clinicians waived third-generation cephalosporins and carbapenems (6 vs. 0 cases). Due to the heterogeneity of this group, containing a mixture of SSTI diagnoses, its statistical power was limited.

### Limitations

Some limitations to this study need to be mentioned. We evaluated main data by overall antibiotic use by RDD/100 BD, because our electronic patient records did not support statistical evaluations by DOT. However, a general trend towards higher RDD/100 BD is seen throughout many German hospitals caused by a progressive reduction of the average amount of time patient spent in hospital. This is due to the German reimbursement system (DRG) and led to a higher consumption per BD (concentrating intravenous antibiotic therapies using maximal doses during the short stay in the hospital). Additionally average age and number of comorbidities increased. More patients with sepsis needed more wide-spectrum antibiotics [39].

Furthermore, a rise of AMS counselling and interventions within the last decade has already had a significant impact on the treatment of *S. aureus* infections such as SAB. This is certainly true for our hospital. Therefore, it´s likely that the advantage of implementing selective reporting might have been shown more clearly in clinics without prior AMS activities.

## Conclusions

This interventional study is, to our knowledge, the first prospective study to prove the impact of selective reporting for *S. aureus* on antibiotic use. There is a strong indication that selective antibiotic reporting improves the therapy of *S. aureus* infections by enhancing the use of narrow-spectrum antibiotics.

Thus, selective reporting of recommended antibiotics is a useful AMS tool, which can be easily implemented with few personnel and technical efforts. We recommend implementing selective reporting rules in the national and international standards for susceptibility reporting.

## Supplementary Information

**Additional file 1**. Flow chart - exclusion criteria of skin and soft tissue infections SSTI.

**Additional file 2**. Flow chart - exclusion criteria S. aureus bacteremia (SAB).

## Data Availability

The datasets used and/or analysed during the current study are available from the corresponding author on reasonable request.

## Abbreviations

BD: Bed days
DOT: Days of therapy
MSSA: Methicillin-sensitive *S. aureus*
MRSA: Methicillin-resistant *S. aureus*
RDD: Recommended daily dose
RDD/100 BD: Recommended daily dose / 100 bed days
SAB: *S. aureus* Bacteremia
SSTI: Skin and soft tissue infections
