# Supplementary figures and images for: Staphylococcus aureus - selective reporting of antibiogram results and its impact on antibiotic use: Interventional study with a reference group on the effect of switching from non-selective to selective antibiotic reporting
Source: Antimicrob Resist Infect Control. 2021 Nov 6;10:157. doi: 10.1186/s13756-021-01021-7 (PMC8572429; doi:10.1186/s13756-021-01021-7)

**Additional file 1** Flow chart - exclusion criteria of skin and soft tissue infections SSTI


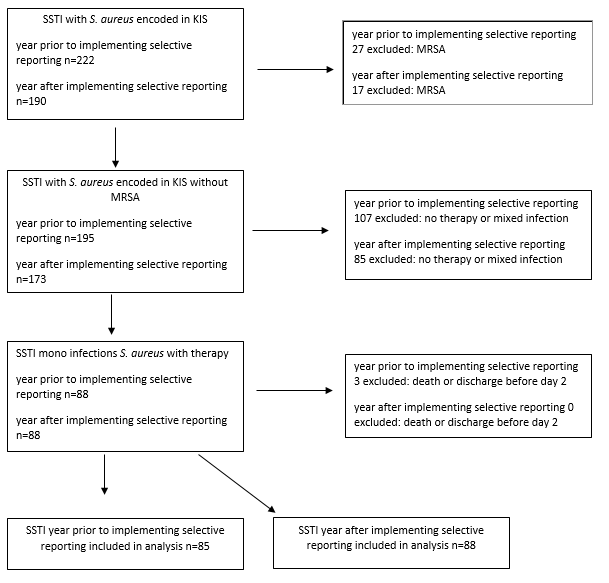

Supplement: Supplementary file 1 — Additional file 1. Flow chart - exclusion criteria of skin and soft tissue infections SSTI. [file 13756_2021_1021_MOESM1_ESM.docx]

**Additional file 2** Flow chart - exclusion criteria *S. aureus* bacteremia (SAB)
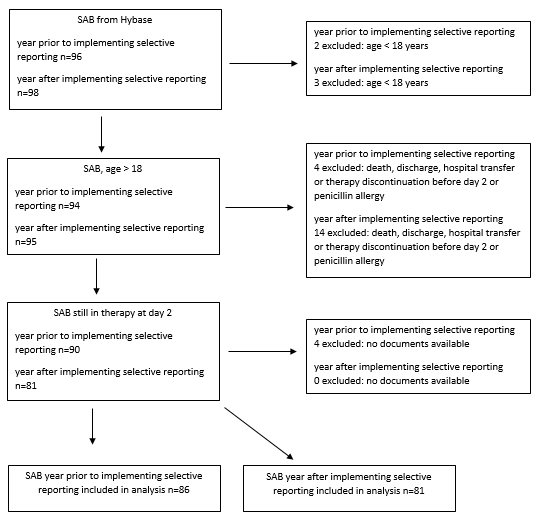

Supplement: Supplementary file 2 — Additional file 2. Flow chart - exclusion criteria S. aureus bacteremia (SAB). [file 13756_2021_1021_MOESM2_ESM.docx]
